# Effects of eating rate on satiety: A role for episodic memory?

**DOI:** 10.1016/j.physbeh.2015.06.038

**Published:** 2015-12-01

**Authors:** Danielle Ferriday, Matthew L. Bosworth, Samantha Lai, Nicolas Godinot, Nathalie Martin, Ashley A. Martin, Peter J. Rogers, Jeffrey M. Brunstrom

**Affiliations:** a Nutrition and Behaviour Unit, School of Experimental Psychology, University of Bristol, UK; b Behavior and Perception Group, Nestlé Research Centre, Switzerland

**Keywords:** Eating rate, Oral processing behaviours, Satiation, Satiety, Episodic memory, Memory for recent eating

## Abstract

Eating slowly is associated with a lower body mass index. However, the underlying mechanism is poorly understood. Here, our objective was to determine whether eating a meal at a slow rate improves episodic memory for the meal and promotes satiety. Participants (*N* = 40) consumed a 400 ml portion of tomato soup at either a fast (1.97 ml/s) or a slow (0.50 ml/s) rate. Appetite ratings were elicited at baseline and at the end of the meal (satiation). Satiety was assessed using; i) an *ad libitum* biscuit ‘taste test’ (3 h after the meal) and ii) appetite ratings (collected 2 h after the meal and after the *ad libitum* snack). Finally, to evaluate episodic memory for the meal, participants self-served the volume of soup that they believed they had consumed earlier (portion size memory) and completed a rating of memory ‘vividness’. Participants who consumed the soup slowly reported a greater increase in fullness, both at the end of the meal and during the inter-meal interval. However, we found little effect of eating rate on subsequent *ad libitum* snack intake. Importantly, after 3 h, participants who ate the soup slowly remembered eating a larger portion. These findings show that eating slowly promotes self-reported satiation and satiety. For the first time, they also suggest that eating rate influences portion size memory. However, eating slowly did not affect ratings of memory vividness and we found little evidence for a relationship between episodic memory and satiety. Therefore, we are unable to conclude that episodic memory mediates effects of eating rate on satiety.

## Highlights

• Eating lunch at a slower rate promotes greater satiation and satiety.
• Participants remember eating a larger meal if they eat it slowly.
• Eating lunch at a slower rate does not affect subsequent snack-food intake.
• Effects of eating rate on satiety might not be mediated by episodic memory.

## 1 Introduction

People consume smaller meals if they eat at a slower pace [1], [2], [3]. By contrast, foods that are eaten quickly tend to be consumed in larger portions [4], [5] and have lower expected satiation [6], [7]. For a recent systematic review and meta-analysis see Robinson et al. [2]. These acute effects are consistent with evidence that faster eating is associated with a higher body mass index (BMI) [8], [9], [10], [11], [12] and that clinically significant (sustained over 12 months) reductions in body weight can be achieved by training obese adolescents to eat slower [13]. Nevertheless, and despite its importance, the underlying causal mechanism that supports a relationship between eating rate and food intake remains poorly understood. In particular, it is unclear how and whether [14] eating rate might influence satiety (the absence of hunger) between meals.

To date, researchers have focused on potential physiological mechanisms. Greater oral processing of a food has been suggested to; i) elicit a stronger cephalic phase response [1], ii) stimulate the release of ‘satiety hormones’ [15], [16], iii) delay gastric emptying [17], and iv) increase lipid bioaccessibility [18]. However, findings relating speed of eating to the release of specific satiety hormones [2], [14] and gastric emptying rate [19] have been inconsistent. Here, we test an alternative (but not mutually exclusive) cognitive explanation. Specifically, we test the hypothesis that eating slowly promotes ‘attentive eating’, which reinforces the encoding of episodic memory for a meal.

There is accumulating evidence that attentive eating and episodic memory play a central role in the control of energy intake [20], [21]. Specifically, it appears that memory (implicit or explicit) of a recent eating episode influences portion selection and energy intake at a subsequent meal. For a recent systematic review and meta-analysis see Robinson et al. [21]. Briefly, it has been noted that amnesic patients demonstrate hyperphagia — they have no memory for a recent meal and experience little change in hunger and fullness shortly after it has been consumed [22], [23]. In neurologically intact participants, there are also converging findings that support an independent role for episodic memory as a determinant of satiety. First, Higgs and colleagues [20], [24], [25], [26] have demonstrated that food intake is reduced if people are asked to recall details of a recent meal. Second, distracting people while they eat has been found to reduce fullness at the end of a meal [27] and to increase food intake at a subsequent meal [28], [29]. Third, attending to the sensory characteristics of a meal reduces intake at a subsequent meal [30], [31]. Finally, in one study the independent roles of episodic memory and gastric feedback were dissociated by manipulating the physical amount of soup that participants consumed relative to the amount they observed [32]. Post-meal hunger was predicted by the remembered rather than the actual portion size, again implicating an important role for episodic memory.

To the authors' knowledge, only one study has explored a causal relationship between oral processing and episodic memory. Specifically, Higgs and Jones [33] showed that increased chewing reduces food intake at a subsequent meal. However, this manipulation had little effect on ratings of memory ‘vividness’. A potential concern is that measures of memory vividness might be dissociable from measures of memory accuracy [34]. In particular, the role of memory for portion size has been implicated [32] and discussed elsewhere [35], [36].

The present study had two objectives. First, we were interested to determine whether eating rate influences fullness at the end of a standard meal and the extent to which this effect is preserved in the inter-meal interval. Participants consumed a fixed portion of soup for lunch. Eating rate was fixed at either a fast or a slow pace. We hypothesised that participants who eat slowly will report greater satiation and greater satiety, and will consume less food at a subsequent snack. Second, we explored evidence that the underlying process is mediated by an effect of eating rate on episodic memory for the lunch. Following a related study [33], we quantified episodic memory using ratings of memory vividness. We also incorporated a novel assessment of memory for portion size.

## 2 Methodology

### 2.1 Participants

Forty participants (20 women and 20 men) were recruited from the staff and student populations of the University of Bristol (United Kingdom) and took part in the study. To reduce demand awareness, participants were told that the purpose of the study was to explore ‘The effects of mood on appetite ratings, taste perception and cognitive performance.’ We excluded participants if they were; i) vegetarian or vegan, ii) not fluent in English, iii) trying to lose weight, iv) taking any medication that might influence appetite or metabolism (with the exception of oral contraceptive pills), or v) allergic or intolerant to any foods. Our sample had a mean age of 23.6 years (*S.D.* = 6.0; range = 18–51) and a mean BMI of 22.8 kg/m^2^ (*S.D.* = 3.4; range = 17.3–32.5). Participants were allocated to either a fast or a slow eating-rate condition (*n* = 20 in each). In remuneration for their assistance, all were offered £15 (Sterling) upon completion of the study. The protocol for the study was approved by the University of Bristol Faculty of Science Human Research Ethics Committee.

### 2.2 Eating rate manipulation

**Fig. 1:**
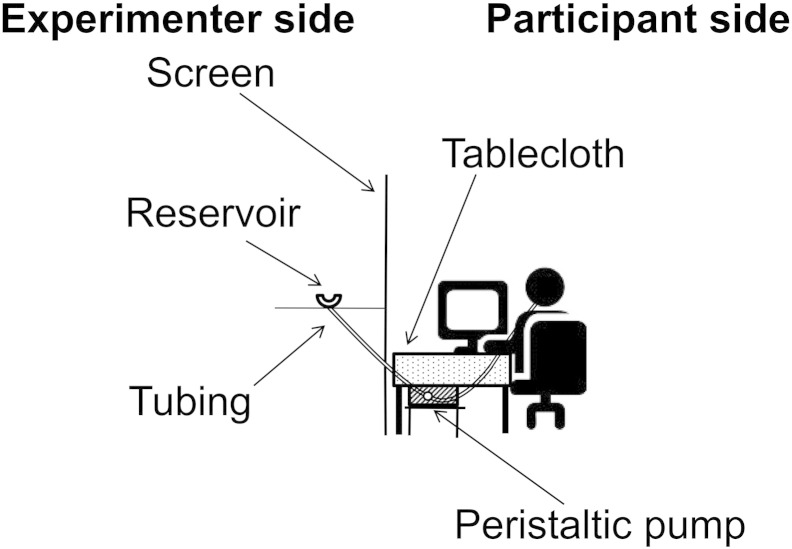
Depiction of the experimental set-up.

Participants consumed a warm tomato soup for lunch (Sainsbury's Supermarkets Ltd, London, U.K.; 59 kcal per 100 ml). Soup was chosen as a test meal because it is at least as satiating as solid foods [37], [38], [39]. To manipulate oral processing, we used a technique that has been employed previously to investigate the effects of sip size and eating rate on *ad libitum* intake [40], [41]. Specifically, the soup was consumed through a temperature-insulated food-grade tube. Participants sat at a table covered by a table cloth. A tall screen was positioned to the left of the participant. The tubing connected to a reservoir of soup (600 ml) via a peristaltic pump (Watson-Marlow, type 323 Du). See Fig. 1 for a depiction of the experimental set-up. Throughout the experiment, the volunteers were unable to see either the pump or the reservoir. Participants were informed that they would be consuming their lunch through a tube because “…people differ in their eating rate, which has been shown to affect people's appetite” and that we were using the pump “…so that everyone eats at the same rate and we can rule out differences that might affect the results.” Each participant consumed 400 ml of soup and the time taken to consume the meal was recorded by the experimenter. To ensure that any effects of eating rate could not be attributed to differences in water intake during the meal [42], participants were given a fixed amount of water with their meal (250 ml) and water intake (g) was recorded.

In the fast eating rate condition, the pump alternated between 2 s of soup delivery (average bite size of 11.8 ml) and 4 s of inactivity. In the slow eating rate condition, 1 s of activity (average bite size of 5.4 ml) was followed by 10 s of inactivity. Note that every time the pump was activated and deactivated it accelerated and decelerated. Across conditions the pump was activated more often in the slow condition, which accounts for the relative difference in flow rate (ml/s).

### 2.3 Taste test

Three hours after lunch, participants took part in a bogus taste test using two different types of biscuits. The procedure for the taste test was identical for all participants. They were presented with two separate 1000 ml clear glass bowls containing ‘custard cream’ biscuits (1000 kcal; 203.3 g) and chocolate chip cookies (1000 kcal; 202.8 g). Biscuits were broken to prevent the participants from counting the number that they had eaten. All foods were supplied by Sainsbury's Supermarkets Limited, Holborn, London. For each type of biscuit, participants were asked to rate five attributes; pleasantness, flavour intensity, sweetness, saltiness, and sourness. Ratings were anchored by ‘not at all’ on the left and ‘extremely’ on the right. Pleasantness ratings were included to establish whether a difference in intake might otherwise be attributed to a differential liking for the biscuits across conditions. Participants were told that any remaining biscuits would be thrown away at the end of the session and that they should feel free to eat as many as they would like. They were not permitted to remove biscuits from the lab at the end of the session. After 10 min, the experimenter returned to the room, removed the biscuits, and the amount consumed (g) was recorded. The amount eaten of each type of biscuit was converted to calories and these values were summed. Participants were also provided with a 250 ml glass of water and water intake (g) was recorded. No other water was made available.

### 2.4 Measures

#### 2.4.1 Appetite and thirst

Participants rated their hunger (Heading: “I feel hungry”; anchor points: “Not at all” and “Extremely”) and fullness (Heading: “My stomach feels full”; anchor points: “Not at all” and “Extremely”) on a computerised 100-mm visual-analogue scale (VAS). From each pair of values, a composite ‘fullness score’ was calculated using the formula ((100 − hunger) + fullness) / 2). Participants also rated their thirst (Heading: “I feel thirsty”; anchor points: “Not at all” and “Extremely”). Appetite and thirst ratings were taken at the beginning of each session, immediately after eating the soup, 2 h after eating the soup, and immediately after the taste test. To reduce demand awareness, these ratings were embedded in other ratings of mood and physical symptoms (MAPS), described below.

#### 2.4.2 MAPS

To be consistent with the cover story (effects of mood on appetite, taste perception and cognitive performance), participants were instructed to rate their MAPS at various times during the experiment. These ratings were based on previous research [43], [44]. Specifically, participants completed computerised 100-mm VAS ratings (Heading: “I feel...” or “My...”; anchor points: “Not at all” and “Extremely”) incorporating the following descriptors; nauseous, tense, mentally alert, heart is racing, hot, physically tired, clear headed, miserable, stressed, friendly, mentally fatigued, relaxed, strange, sleepy, energetic, head aches, able to take on a physically demanding task, able to concentrate, angry, lethargic, and cheerful.

#### 2.4.3 Episodic memory for food

To assess episodic memory for the lunch, we included two measures. First, based on previous research [28], [33], participants were asked to rate “How vividly do you remember the lunch you ate earlier today?” (anchor points: “Not at all” and “Extremely”). Second, based on previous studies [32], [41], participants were given an empty clear bowl and an identical bowl containing 1200 ml of soup. They were then asked to serve into the empty bowl the volume of soup that they believed they had consumed earlier. For each participant, an index of memory accuracy was calculated by subtracting the amount that was actually consumed (400 ml) from the amount that participants believed that they had consumed. The order of these tasks was counterbalanced across participants.

#### 2.4.4 Self-reported eating rate

Based on previous research [45], participants were asked “How would you describe your usual rate of eating?” They answered on a 5-point qualitative scale, with the categories; ‘Very slow’, ‘Relatively slow’, ‘Medium’, ‘Relatively fast’, and ‘Very fast.’ Following Petty et al. [45], w*e* classified participants into one of three categories (slow, medium, and fast eaters) based on their responses; the ‘very slow’ and ‘relatively slow’ categories were combined, as were the ‘very fast’ and ‘relatively fast’ responses. This enabled us to assess evidence for between-group differences (slow *versus* fast eating-rate conditions) in typical eating rate.

### 2.5 Procedure

Test sessions were scheduled every weekday between 12:00 and 14:00 in the Nutrition and Behaviour Unit. Participants were instructed to consume their normal breakfast and to abstain from eating and consuming calorie-containing beverages for at least 3 h prior to their appointment. On arrival they read an information sheet and signed a consent form. They then confirmed compliance with our request to abstain from eating and provided details of their most recent meal.

Participants then completed baseline ratings of appetite (hunger and fullness), thirst and MAPS. Afterwards, they consumed the soup at either the fast or slow rate (dependent on condition allocation). Immediately after eating, participants completed a second set of ratings. They were then allowed to leave the laboratory but were instructed to abstain from eating and from drinking calorie-containing beverages. After 120 min, the participants returned and completed a third set of ratings. They then spent 60 min completing various cognitive tasks that included word-pair learning. These were incorporated to be consistent with the cover story (effect of mood on cognitive performance) and associated data are not presented. Participants then took part in the biscuit taste test to assess *ad libitum* snack food intake. Immediately afterwards they provided a final set of ratings. Participants were then asked to complete the episodic memory measures, followed by the Dutch Eating Behaviour Questionnaire (DEBQ) [46]. To assess the extent to which our participants were aware of the aims of the study, they were asked to provide a written response to the question, “Please describe in as much detail as possible what you believe the aim of this study was?” Finally, participants reported their usual rate of eating and their weight, height and age were recorded. Debriefing took place by email, after all of the data had been collected.

### 2.6 Data analysis

In the first instance, the raw data were converted to *z*-scores and screened for outliers. Scores falling outside 99.9% of a normal distribution were entered as missing data [47]. All data were analysed using IBM SPSS statistics version 21 (IBM, New York, USA). In all analyses we applied a critical *p*-value of <.05.

To explore across-condition differences in baseline measures and participant characteristics, we used independent samples *t*-tests. Analyses were performed on time since last eating, baseline fullness, baseline thirst, BMI, age, DEBQ restraint, DEBQ external eating, and DEBQ emotional eating. A chi-square test was used to assess differences in the number of self-reported fast, medium, and slow eaters allocated to each condition. *Post hoc*, we were concerned that our manipulation of eating rate might also impact mood [33], which is found to impact appetite directly [48], [49]. Therefore, to evaluate evidence for this alternative account we used separate multivariate ANOVAs (gender was included as a between-subjects factor) to assess the main effect of eating rate on MAPS, at baseline and immediately after lunch.

To assess the efficacy of our manipulation of eating rate, for each participant, the amount of soup consumed (400 ml) was divided by the recorded meal duration (s). An independent samples *t*-test was then used to explore across-condition differences in eating rate (ml/s).

Change from baseline fullness scores were calculated by subtracting baseline fullness ratings from post-meal ratings. Separate scores were calculated for each participant and each post-meal rating (immediately post-lunch, pre-snack, and post-snack). A 2 (eating rate; fast or slow) × 2 (gender; male or female) between-subjects ANOVA was used to explore effects of eating rate on change from baseline fullness, immediately after lunch (satiation). To explore effects of our eating rate manipulation on self-reported satiety, we conducted a mixed-measures ANOVA. Time (2 levels; 2 h after lunch and after the taste test) was the within-subjects factor. Condition (2 levels; slow or fast) and gender (2 levels; male or female) were between-subjects factors. To analyse the thirst ratings, similar change from baseline scores were calculated as outlined above and were submitted to identical ANOVAs.

A 2 (eating rate; fast or slow) × 2 (gender; male or female) between-subjects ANOVA was then used to assess the effect of eating rate condition on; i) snack intake, ii) biscuit sensory ratings, iii) water intake, iv) memory vividness, and v) portion size memory. In all analyses, no significant interactions with gender were observed, so combined group means are reported.

Finally, to evaluate whether effects of eating rate on satiety might be mediated by differences in episodic memory, we correlated (Pearson's *r*) our measures of episodic memory (portion size memory and vividness) and our assessments of satiety (self-reported fullness and biscuit intake).

## 3 Results

### 3.1 Baseline measures and participant characteristics

Table 1 shows the means (and associated standard deviations) for baseline scores of appetite, thirst, and participant characteristics, in the fast and slow eating rate conditions, separately. At baseline, participants in the two conditions did not differ significantly in their time since last eating, fullness, thirst, BMI, age, or DEBQ subscale scores. There was also an equal number of self-reported slow, medium and fast eaters in each condition. Analysis of the MAPS ratings at baseline revealed no statistically significant differences between conditions (all *p* ≥ .07; see Table 2), with the exception of ratings of mental fatigue; participants in the slow condition reported greater mental fatigue than those in the fast condition (*F*(1, 34) = 5.27, *p* = .03).

### 3.2 Effect of the eating rate manipulation on meal duration and water intake

The average recorded meal duration for participants in the fast eating condition was 203 s (*S.D.* = 4 s), whereas in the slow condition the average meal duration was 800 s (*S.D.* = 18 s). This generated an average eating rate (ml/s) of 0.50 (*S.D.* = 0.01) and 1.97 (*S.D.* = 0.04) in the slow and fast conditions, respectively. This is almost a four-fold difference and is highly significant (*t*(38) = 153.9, *p* < .001). Eating the soup at different rates did not affect the volume of water consumed during the meal (see Table 3).

### 3.3 Appetite, thirst and MAPS ratings immediately after lunch

**Fig. 2:**
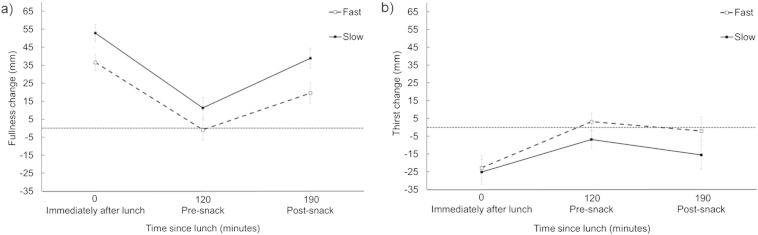
Mean (± *S.E.M.*) change in fullness composite scores (panel a) and thirst ratings (panel b) immediately after lunch, at 120 min (before the taste test), and at 190 min (after the taste test). Results are reported separately for the fast and slow eating conditions. The dotted horizontal line represents rated appetite or thirst before consuming the soup (baseline).

Immediately after eating the soup, participants who consumed it at the slow rate reported a significantly greater change in fullness from baseline (*F*(1, 36) = 6.28, *p* = .02; see Fig. 2). There were no significant differences in change in thirst from baseline (*F*(1, 36) = 0.06, *p* = .81; see Fig. 2) or any of the MAPS ratings between conditions (all *p* ≥ .05; see Table 2), with the exception of strangeness; participants in the fast condition reported greater strangeness at the end of the meal (*F*(1, 33) = 4.67, *p* = .04).

### 3.4 Appetite and thirst ratings in the inter-meal interval

We then considered whether the effects of eating rate extend beyond the end of the meal and are preserved at 120 min and 190 min (before and after the taste test). Our analysis showed that participants who consumed the soup at a slow rate reported a significantly greater change in fullness from baseline (*F*(1, 36) = 5.49, *p* = .03; see Fig. 2). The interaction between eating rate condition (slow and fast) and time (120 and 190 min) failed to reach significance (*F*(1, 36) = 0.76, *p* = .39). There was no significant difference in rated thirst 120 min and 190 min after eating the soup (*F*(1, 36) = 2.08, *p* = .16; see Fig. 2).

### 3.5 Snack intake, sensory ratings, and water intake

Eating rate at lunchtime did not affect *ad libitum* intake in the subsequent taste test (see Table 3). There was also no difference in water intake during the taste test between conditions (see Table 3) and no significant differences in any of the sensory ratings of the biscuits between conditions (all *p* > .16).

### 3.6 Episodic memory for food

Participants who consumed the soup at the slow rate were significantly less accurate in remembering their portion size — they recalled eating a larger portion 3 h later (see Table 3). There was also a trend towards participants in the slow condition reporting a more vivid memory for the soup. However, the associated test failed to reach significance (see Table 3). *Post-hoc*, we recognised that participants in the slow condition reported greater satiety at the same time that the memory measures were completed. In response to a concern about a potential confound, we repeated our analyses with the 190-minute composite fullness scores included as a covariate. The results remained unaltered.

### 3.7 Correlations between measures of satiety and episodic memory

Table 4 summarises the correlations between our measures of episodic memory (portion size memory and vividness) and our assessments of satiety (change in self-reported fullness and snack intake). Change from baseline fullness at 120 min and 190 min after lunch were positively correlated. There was also a positive relationship between portion size memory accuracy and memory vividness. However, this relationship failed to reach significance (*p* = .10). The relationships between satiety and episodic memory were all non-significant and had a small or trivial effect size.

### 3.8 Demand awareness

In response to the open-ended question about the purpose of the study, 80% of the participants believed the cover story and suggested that the study was investigating the inter-relationships between appetite, mood and cognitive performance (*e.g.*, concentration and attention). The remaining 20% offered alternative suggestions, none of which related to the objectives of the study.

## 4 Discussion

This study sought to address two questions. First, we were interested to determine whether eating rate influences fullness at the end of a standard meal and the extent to which this effect is preserved in the inter-meal interval. Second, we explored evidence that this process is accompanied by differential effects of eating rate on episodic memory. The following sections consider the extent to which these questions can now be answered.

### 4.1 Effects of eating rate on satiation and satiety

Immediately after consuming the soup, we found that eating at a slower rate was associated with a greater increase in self-reported fullness (Cohen's *d* estimate of effect size = 0.78). This is consistent with our hypothesis and with previous research showing that satiation can be enhanced by eating at a slower rate [50] or by modifying the texture of food [40], [51], [52] (see Robinson et al. [2] for a review). Several studies have assessed relationships between self-reported appetite and subsequent energy intake [53], [54], [55], [56]. One suggestion is that a 15 to 25 mm difference (on a 100-mm VAS scale) in rated fullness is sufficient to correspond with a reliable difference in *ad libitum* food intake [57]. In the present study, eating at a slower rate increased our observed change in fullness by 16 mm, suggesting that a meaningful difference was achieved. Consistent with a previous study [42], this effect was observed despite the fact that water intake was matched across conditions. In addition, this effect does not appear to be mediated by differences in mood. In our *post-hoc* analysis eating rate influenced only one of the 21 post-meal MAPS ratings — participants in the fast condition reported feeling significantly more ‘strange’. We suspect this difference is spurious, it was marginal, and would not survive a correction for multiple tests.

In participants who ate at the slow rate, we observed a greater increase in self-reported satiety 2 h after the meal (Cohen's *d* estimate of effect size = 0.47) and also after the taste test (Cohen's *d* estimate of effect size = 0.77). These results complement our previous study showing that satiety is increased when a fixed-portion meal is eaten slowly [7]. By contrast, we did not see an effect of eating rate on intake in the subsequent taste test. It is unlikely that we were underpowered to detect this effect for three main reasons. First, our sample size (*n* = 20 per condition) exceeds other relevant research which has observed a significant effect of manipulating number of chews-per-mouthful on subsequent snack intake [33]. Second, according to widely accepted recommendations regarding effect sizes [58], the effect size observed on this measure was trivial (Cohen's *d* estimate of effect size = 0.10). Third, a selective effect of eating rate on self-reported satiety but not subsequent food intake has been reported previously [17], [59], [60]. In this regard, we note that appetite ratings often reflect a motivation to begin an eating episode but may be a relatively poor predictor of the amount consumed [61], [62]. From this, it follows that faster eaters may be more likely to consume a greater number of meals (rather than larger meals) and it would be interesting to determine whether this accounts for previously observed relationships between BMI and eating rate [10], [11].

An unexpected finding was that the effects of eating rate on self-reported satiety were evident even after the participants had completed the taste test. One explanation for this finding is that participants might have treated the taste test literally and not eaten *ad libitum* (*i.e.*, consumed only ‘taster portions’ in order to complete sensory ratings). Consistent with this idea, after the taste test, participants reported only modest fullness (fast: M = 56.3 mm, slow: M = 65.8 mm; 100 mm VAS scale). We recommend that before discounting an effect of eating rate on subsequent *ad libitum* meal size, future studies should address our concern by instructing participants to eat until they are comfortably full.

### 4.2 Effects of eating rate on episodic memory

Our manipulation of eating rate also affected episodic memory for the lunch during the inter-meal interval. We hypothesised that participants in the slow condition would demonstrate enhanced memory for the lunch. Contrary to our hypothesis, eating at a slower rate was associated with a less accurate portion size memory after 3 h. However, and importantly, participants in the slow eating rate condition remembered eating a significantly larger portion (Cohen's *d* estimate of effect size = 0.65). This finding is consistent with a recent study demonstrating that consuming soup *ad libitum* and with smaller sip sizes leads to an overestimation of the amount consumed, when assessed immediately after the meal [41]. For the first time, our finding suggests that this effect might be remembered and preserved during the inter-meal interval (3 h later).

For now, it remains unclear how eating rate affected portion size memory. During the lunch, participants consumed the soup through a tube and were unable to see the soup reservoir. In this unusual context, participants might be biased by differences in perceived meal duration or otherwise by an implicit process associated with the metering of oral exposure (governed by factors such as the number and volume of sips). Alternatively, their estimate may be based on a memory of fullness, immediately after consuming the meal. To explore the role of visual cues of amount eaten, future research should attempt to replicate the effect of eating rate on portion size memory when the reservoir of soup is visible to participants.

We also assessed memory quality using ratings of self-reported memory vividness, a measure that has been used widely in previous research [29], [33]. Manipulating eating rate did not affect the rated vividness of memory for lunch. This is consistent with a recent observation that increased chewing does not affect vividness [33]. It may be relevant that measures of memory vividness provide a proxy for an individual's confidence or belief in their memory rather than its accuracy — particularly for emotional memories [34]. We also note that there was a non-significant trend for greater vividness in the slow condition (Cohen's *d* estimate of effect size = 0.48), suggesting that we were underpowered to detect an effect of eating rate (a sample size of 108 would be required to detect this effect with an α of 0.05 and a 1 − β of 0.80).

### 4.3 Evaluation of the eating rate and episodic memory hypothesis — summary

A critical test of the eating rate and episodic memory hypothesis would be an analysis of whether differences in episodic memory mediate the relationship between eating rate and satiety. In this regard, we note that we did observe independent relationships between eating rate and satiety, and between eating rate and portion size memory. However, episodic memory (vividness and portion size memory) and satiety (self-reported fullness and biscuit intake) were unrelated, suggesting a lack of causation.

However, before rejecting the eating rate and memory hypothesis, we note that eating a meal is complex and it has the potential to generate a rich set of memories associated with orosensory, visual, and visceral experiences. Some of these may still play an important role but were not captured by our assessments of vividness and portion size memory. Future research might develop and include additional measures of memory accuracy. In particular, they might consider measures of taste memory [63], colour memory [64], and memory for the order of food items consumed in a meal [29].

### 4.4 Episodic memory measures and satiety — general implications

Three hours after consuming the soup meal, a significant difference was observed in portion size memory between the fast and slow conditions (approximately 108.8 ml). Previously, comparable differences in portion size memory (approximately 125.6 ml) have been shown to be an important predictor of self-reported satiety [32]. Given this, it is perhaps surprising that the difference in portion size memory observed in this study was not associated with differences in self-reported satiety or *ad libitum* snack intake. While we acknowledge that there are many explanations for a null finding, the effect sizes observed in the present study were small/trivial suggesting that our failure to find a relationship is not due to a lack of statistical power. It may be relevant that participants consumed the soup through a tube and the reservoir of soup was not visible to them. In this unusual situation, participants may have been uncertain about the amount of soup that they had consumed during the meal, felt less confident in their portion size memory during the inter-meal interval, and might have relied on other cues instead (*e.g.*, gastric distension) when making their appetite ratings. For now, this proposition is speculative and remains to be tested empirically.

More generally, many studies have demonstrated an effect of manipulations of attentive eating on measures of episodic memory (for a review, see Robinson *et al.* [21]). However, to the authors' knowledge only four studies have directly assessed the relationship between measures of episodic memory for a recently consumed meal and subsequent *ad libitum* intake. Of these studies, two reported a moderate significant negative correlation [29], [33] and two report no relationship [31], [65]. In light of these findings, and the observations that we report here, it may be premature to claim a causal role for memory processes in the control of food intake from meal to meal.

## Figures and Tables

**Table 1:** Mean (± *S.D.*) scores of time since last eating (min), baseline fullness (mm), baseline thirst (mm), BMI (kg/m^2^), age (years), DEBQ restraint, DEBQ external eating, and DEBQ emotional eating, in the fast and slow eating rate conditions, separately. The number of self-reported slow, medium and fast eaters in each condition is also displayed.

	Fast eating rate (*n* = 20)	Slow eating rate (*n* = 20)	*t*(38)	*p*
Time since last eating (min)	307.0 (277.8)	355.5 (215.2)	− 0.6	.54
Baseline fullness (mm)	36.7 (21.8)	26.9 (13.3)	1.7	.09
Baseline thirst (mm)	48.5 (21.0)	52.4 (25.0)	− 0.5	.60
BMI (kg/m^2^)	22.5 (3.2)	23.1 (3.6)	− 0.6	.57
Age (years)	23.7 (7.5)	23.6 (4.2)	0.1	.96
DEBQ restraint	2.3 (0.8)	2.4 (0.8)	− 0.6	.56
DEBQ external eating	3.7 (0.5)	3.6 (0.6)	0.5	.65
DEBQ emotional eating	2.7 (0.8)	2.6 (0.9)	0.7	.46
Self-reported eating rate	3 slow/6 medium/11 fast	3 slow/6 medium/11 fast	*X*^2^ = .00	1.00

**Table 2:** Mean (± *S.D.*) ratings (mm) of nausea, tense, mentally alert, heart is racing, hot, physically tired, clear headed, miserable, stressed, friendly, mentally fatigued, relaxed, strange, sleepy, energetic, head aches, able to take on a physically demanding task, able to concentrate, angry, lethargic and cheerful. Results are shown for ratings obtained at baseline and immediately after lunch and are reported separately for the slow and fast conditions.

Rating (mm)	Fast eating rate (*n* = 20)		Slow eating rate (*n* = 20)	
Baseline	Post-lunch	Baseline	Post-lunch
Nauseous	12.4 (19.3)	23.8 (23.9)	10.8 (15.5)	16.8 (22.0)
Tense	19.4 (20.9)	16.9 (17.1)	19.4 (25.4)	10.5 (17.0)
Mentally alert	53.1 (24.7)	52.6 (25.3)	44.4 (19.3)	49.6 (27.4)
Heart is racing	7.9 (10.9)	9.5 (12.4)	20.2 (26.5)	10.9 (13.7)
Hot	19.5 (20.2)	18.7 (20.8)	23.8 (28.0)	19.9 (22.0)
Physically tired	33.7 (26.5)	21.1 (17.3)	40.7 (25.8)	32.9 (21.0)
Clear headed	56.4 (25.8)	58.7 (21.9)	54.9 (21.9)	55.7 (23.0)
Miserable	10.7 (17.7)	12.8 (13.9)	7.6 (8.2)	6.6 (9.1)
Stressed	20.8 (22.0)	17.3 (19.4)	28.1 (27.3)	24.9 (23.5)
Friendly	58.4 (13.8)	60.0 (15.9)	55.6 (22.7)	52.5 (23.9)
Mentally fatigued	31.2 (23.0)	27.3 (23.1)	49.2 (24.8)	32.9 (25.2)
Relaxed	63.6 (16.3)	63.7 (16.3)	55.9 (21.3)	64.9 (24.2)
Strange	17.0 (21.7)	15.8 (16.1)	20.9 (20.3)	7.0 (7.7)
Sleepy	35.4 (25.7)	30.9 (21.8)	43.3 (27.6)	41.4 (23.0)
Energetic	39.7 (20.2)	47.3 (20.5)	40.3 (24.4)	43.4 (21.9)
Head aches	8.9 (14.4)	8.1 (10.8)	18.4 (23.3)	8.3 (10.0)
Able to take on a physically demanding task	52.8 (23.0)	50.0 (29.3)	51.3 (27.6)	59.8 (23.5)
Able to concentrate	61.6 (18.6)	63.5 (18.9)	56.0 (23.5)	56.9 (21.3)
Angry	10.9 (18.1)	9.1 (11.4)	12.9 (17.7)	6.4 (11.2)
Lethargic	32.3 (24.3)	30.0 (21.5)	46.9 (26.9)	40.7 (23.1)
Cheerful	52.8 (17.4)	55.2 (17.0)	54.8 (23.9)	57.1 (21.7)

**Table 3:** Mean (± *S.D.*) scores for water intake (g), snack intake (kcal), portion size memory accuracy (ml)^a^ and ratings of memory vividness for lunch (mm), in the fast and slow eating rate conditions, separately.

	Fast eating rate (*n* = 20)	Slow eating rate (*n* = 20)	Main effect of eating rate *F*(1, 36)	*p*
*Water intake* (*g*)				
During lunch	89.3 (57.7)	104.4 (74.3)	0.49	.49
During taste test	161.0 (75.3)	166.6 (72.7)	0.06	.82
*Snack intake* (*kcal*)	505.5 (252.1)	484.0 (198.0)	0.13	.72
*Food memory measures*				
Portion size memory accuracy (ml)^a^	− 32.3 (118.8)	76.5 (204.8)	4.14	.049
Memory vividness (mm)	66.1 (20.0)	76.6 (23.2)	2.31	.14

a Calculated by subtracting the amount that was actually consumed (400 ml) from the amount that participants believed that they had consumed.

**Table 4:** Correlations (Pearson's *r*) between self-reported satiety, *ad libitum* snack intake, portion size memory accuracy and memory vividness (**p < .01).

	Change in fullness (mm) at 120 min (before taste test)	Change in fullness (mm) at 190 min (after taste test)	*Ad libitum* snack intake (kcal)	Portion size memory accuracy (ml)	Memory vividness (mm)
Change in fullness (mm) at 120 min (before taste test)	–				
Change in fullness (mm) at 190 min (after taste test)	.50**	–			
*Ad libitum* snack intake (kcal)	−.17	−.04	–		
Portion size memory accuracy (ml)	−.12	−.09	.03	–	
Memory vividness (mm)	.08	.14	.07	.26	–
